# EVALUATION OF RISK FACTORS AFFECTING ANASTOMOTIC LEAKAGE AFTER REPAIR OF ESOPHAGEAL ATRESIA

**DOI:** 10.1590/S0102-67202015000300003

**Published:** 2015

**Authors:** Shahnam ASKARPOUR, Mehran PEYVASTEH, Hazhir JAVAHERIZADEH, Nasim ASKARI

**Affiliations:** From the Department of Pediatric Surgery, Imam Khomeini Hospital, Ahvaz Jundishapur University of Medical Sciences, Ahvaz, Iran

**Keywords:** Anastomosis, Anastomotic leak, Esophageal atresia

## Abstract

**Background:**

Anastomotic leak are reported among neonates who underwent esophageal atresia.

**Aim:**

To find risk factors of anastomotic leakage in patients underwent esophageal repair.

**Methods:**

All cases with esophageal atresia were included. In this case control study, patients were classified in two groups according to presence or absence of anastomotic leaks. Duration of study was 10 years.

**Results:**

Sixty-one cases were included. Mean±SD age at time of surgery in patients with leakage and without leakage was 9.50±7.25 and 8.83±6.93 respectively (p=.670). Blood transfusion and two layer anastomosis had significant correlation with anastomotic leakage.

**Conclusion:**

Blood transfusion and double layer anastomosis are associated with higher rate of anastomotic leakage.

## Abstract (pt)

**Racional:**

Fístulas são relatadas entre os recém-nascidos que foram submetidos à reparação de atresia de esôfago.

**Objetivo:**

Encontrar fatores de risco de deiscência de anastomose nos pacientes submetidos à correção cirúrgica do esôfago.

**Métodos:**

Todos os casos com atresia de esôfago foram incluídos. Neste estudo caso-controle, os pacientes foram classificados em dois grupos de acordo com a presença ou ausência de fístula. Duração do estudo foi de 10 anos.

**Resultados:**

Sessenta e um casos foram incluídos. A média±DP da idade no momento da operação em pacientes com deiscência e sem foi 9,50±7,25 e 8,83±6,93, respectivamente (p=0,670). Transfusão de sangue e duas camadas anastomose tiveram correlação significativa com a deiscência da anastomose.

**Conclusão:**

Transfusão de sangue e anastomose em dois planos estão associadas com maior taxa de deiscência.

## INTRODUCTION

The overall incidence of esophageal atresia ranges from 1/2500 to 1/4000 live births. Anastomotic leaks occur in 15-20% of patients. Several factors may play a role in pathophysiology of leaks such as: friable lower segment, ischemia of the esophageal ends, sepsis, poor suturing techniques, the type of suture, and excess anastomotic tension 12 . Anastomotic leakage may be associated with some complications. Peyvasteh et al. 8 showed incidence of stricture significantly higher in patients who developed anastomosis leak after repair^2^. There are few published papers about anastomotic leakage after repair of esophageal atresia. 

The aim of this study was to find possible factors which may play a role in anastomotic leaks after surgical repair of esophageal atresia.

## METHODS

This study was realized at the Department of Pediatric Surgery of Imam Khomeini Hospital of Ahvaz Jundishapur, University of Medical Sciences, Ahvaz, Iran, and was approved by ethical committee of the university

All cases with esophageal atresia were included. Gross classification was used for classification of esophageal atresia. In this case control study, patients were classified in two groups: with and without anastomotic leakage. 

SPSS version 13.0 (Chicago, IL, USA) was used for data analysis. t-Test and Chi-Square were used for comparison. 

## RESULTS

Sixty-one cases were included. Mean age±SD at the time of surgery in patients with anastomotic leakage and without was 9.50±7.25 and 8.83±6.93 days, respectively (p=0.670). 

**TABLE 1:** Analysis of factors among children with and without leakage

p	Without Leakage (n=48)	With Leakage n=13
0.363	26(83.9%) 22(73.3%)	5(16.1%) 8(26.7%)	F M	Sex
0.023	18(94.7%) 28(75.7%) 2(40%)	1(5.3%) 9(24.3%) 3(60%)	0 1 2	Blood transfusion
0.016	46(83.6%) 2(33.3%)	9(16.4%) 4(66.7%)	One Layer Two Layer	Technique of anastomose
0.547	3(60.0%) 3(75.0%) 42(80.8%)	2(40.0%) 1(25.0%) 10(19.2%)	A B C	Type of atresia
0.131	9(64.3%) 39(83.0%)	5(35.7%) 8(17.0%)	Long Short	Gap length
0.209	37(75.5%) 11(91.7%)	12(24.5%) 1(8.3%)	Extrapleural Intrapleural	Thoracotomy type
0.209	37(75.5%) 11(91.7%)	12(24.5%) 1(8.3%)	Yes No	Pre-operation pneumonia
0.164	12.95±4.35	15.023±4.60	Pre-operation WBC
0.84	14.07±2.36	13.90±2.75	Pre-operation Hb
0.488	7.48±3.02	8.92±7.12	Starting oral feeding after surgery (day)
0.7	6.62±4.75	6.08±4.85	Pre-operation hospitalization time (day)
.29	8.83±6.93	9.50±7.22	Age at operation (day)
0.5	2865±379	2755±710	Body weightv(gram)

Anastomotic leakage had positive correlation with blood transfusion (p=0.023) and two layer anastomosis (p=0.016). There were no significant differences between both groups in terms of gender, type of atresia, gap length, pre-operation hemoglobin and pre-operation pneumonia ( Table 1 )

## DISCUSSION

In this study of 61 cases, 13 (21%) had anastomotic leakage. In Peyvasteh et al. ^3^study from Tehran, Iran, anastomotic leakage was reported in 18 (24.32%) of 74 patients. Chittmittrapap et al. 3 reported that anastmosis leakage was present in 17% of cases. In Konkin et al. 4 study, anastomotic leakage was reported in 8% of cases. Anastomotic leakage was reported about 15-20% in other studies 5 6 9 10 11 . Anastomotic leakage rate of this study was similar to other reports.

Incidence of esophageal leak had significant difference according to atresia type 12 . But in this study, there was no significant difference. There was no relationship between the type of thoracotomy and leakage of anastomosis. This is similar to Askarpour et al. 1 report. 

Double layer anastomosis was significantly associated with anastomotic leakage. In Aslam et al. 2 paper, was related no difference between single versus double layer anastomosis regarding leakage.

Limitations of this report are mainly connected on being a retrospective analysis and, also, realized in a single center.

## CONCLUSION

Blood transfusion and two layer anastomosis were associated with anastomotic leakage in esophageal atresia in children
